# Hematological and Biochemical Reference Intervals for Mules in Chile

**DOI:** 10.3389/fvets.2019.00400

**Published:** 2019-11-12

**Authors:** Javiera Lagos, Tamara A. Tadich

**Affiliations:** ^1^Programa de Doctorado en Ciencias Silvoagropecuarias y Veterinarias, Campus Sur, Universidad de Chile, Santiago, Chile; ^2^Departamento de Fomento de la Producción Animal, Facultad de Ciencias Veterinarias y Pecuarias, Universidad de Chile, Santiago, Chile

**Keywords:** mules, blood reference intervals, hematology, biochemistry, working equids

## Abstract

Hematological and biochemical reference intervals are an important tool for health assessment and treatment decision-making in veterinary medicine. Lack of information about reference intervals (RI) in mules force professionals to apply reference intervals developed for horses or donkeys, with the risk of misinterpretation. Thus, the aim of this study was to determine hematological and biochemical RI for healthy mules and compare them with those proposed in literature for horses, donkeys and mules. A total of 142 healthy mules (mixed breed mares × Baudet du Poitou) of both sex, all between 7 and 22 years of age and between 290 and 500 kg of live weight were sampled and 32 blood parameters analyzed in order to calculate reference intervals according to the International Federation of Clinical Chemistry and Laboratory Medicine (IFCC) and the Clinical Laboratory Standards Institute (CLSI) standards. The values obtained for Chilean mules were within the RI in three of the 21 variables where data was available for UK donkeys and for three of 22 RI available for working horses in Pakistan; no similarities were found with those from Portuguese mules. In the case of Chilean working horses RI, mules values were within range for 11 of 25 variables. The differences found in blood biochemistry may be explained by husbandry conditions, diet, type of work and biological features. Differences between mules' reference intervals and those from donkeys and working horses highlight the importance of having specific reference values for this equid hybrid and the need to develop further research in mules under different working conditions and genetic background.

## Introduction

Hematological and biochemical reference intervals (RIs) play a fundamental role for assessing the health condition, physiologic alterations, disease diagnosis and treatment decision-making in animals; at the same time they provide a very useful tool for animal welfare evaluations (1, 2). A key aspect of blood reference intervals is that they are influenced by multiple factors, such as genetics, breeds, husbandry practices, and environmental conditions (3, 4). Previous studies have found important differences in hematological and biochemical values when comparing the reference intervals of a general population and those obtained for specific breeds or local populations, highlighting the risk of an inappropriate use of RI when conducting clinical or research studies (4–6).

Mules are widely used for transport of people and their supplies, especially in difficult mountainous terrains where vehicles' access is difficult or even impossible (7). On the other hand, they are used for endurance equestrian sport because of their physical strength (8). However, the lack of blood reference intervals for this hybrid hinders the assessment of the effect of exercise intensity on musculature, blood electrolytes and hematological parameters. Thus, there is a risk for sport and working mules to be overworked and misdiagnosed (6) and therefore, of compromising their welfare state.

A considerable amount of literature has been published about horses RIs of different breeds and functions (2, 6, 9). Aros et al. (4) found important differences between hematological and biochemical parameters established for working horses in Chile and those obtained for sport horses, while in contrast Chilean working horses' RIs overlapped better with those obtained for working horses in Pakistan. Leidinger et al. (10) calculated RI for the Icelandic horse breed, finding that several parameters differed from the RI commonly used for horses. In the same line, some research has evaluated RI in donkeys, showing important differences with data provided for horses (3, 5). In the case of mules, to our knowledge only two studies have attempted to establish their blood RI (9, 11). These studies involved small samples (*n* = 20 in both studies) and particular local conditions; nevertheless, they were able to observe important differences between values obtained for mules and those obtained for horses and donkeys.

Therefore, despite the great support that mules provide as pack animals, for tourism and for equestrian sports, there is still a lack of knowledge about normal hematological and biochemical values that would allow more precise disease diagnoses and welfare assessments for these hybrids. This is why, the aim of this study was to determine hematological and biochemical reference intervals for healthy mules and compare them with those proposed in the literature for horses, donkeys, and mules.

## Materials and Methods

### Animals

All measurements were taken from a reference Chilean population of mules that belongs to the army. The selection criteria proposed by Pritchard et al. (6) for working horses was applied, this means that a healthy mule was one that had no clinical signs of disease after performing a complete clinical examination. In addition, only mules with a body condition score (BCS) between 2 and 4 in a 5 point BCS system and that had been free of any type of medication during the 7 days preceding the sampling were included. A total of 142 mules met the inclusion selection criteria and were sampled. From these, 83 were mares and 59 geldings, all aged between 7 and 22 years, bodyweights between 290 and 500 kg. The genetic background of these mules is the cross of mix breed mares with Baudet du Poitou and local cross breeds for jacks. All mules are owned by the Chilean army, 119 belonged to the Detachment N°3 “Yungay” and 23 from the Chilean Army Mountain School, both located in the urban and peri-urban areas of the city of Los Andes, in the central area of the country. The main purpose of the Chilean army mules is the transport of supplies and materials to mountain areas, otherwise of difficult access. Mules are fed with 6 kg of alfalfa hay twice a day, water is provided *ad libitum* and are kept in paddocks, only if weather conditions are adverse during winter they are housed in a loose system (barn).

### Hematological and Biochemical Variables

Blood samples were collected by jugular venipuncture during mules' resting days, using a 20 ml syringe and 21G needle. Blood was then transferred into three tubes (4 ml with EDTA, 8 ml with no additive and 4 ml with heparin). Samples were stored in ice in a cooler box and transported to external services for processing within the first 24 h of collection.

For hematology, hemoglobin [Cyanomethemoglobin method using Hitachi^®^, Photometer 4020 (Boehringer Mannhein)], hematocrit, red blood cells (RBC) count, mean corpuscle volume (MCV), mean corpuscular hemoglobin concentration (MCHC), erythrocyte sedimentation rate (ESR), white blood cell (WBC) count, neutrophils (N), lymphocytes (L), neutrophils-lymphocytes ratio (N:L), monocytes, eosinophils, basophils, and platelets were determined (Abacus Junior Vet^®^). Differential leucocyte counts and erythrocyte morphology were performed on blood-stained smears using a Romanowski stain (Corzap 1, Hemogram^®^) at 1,000 × (Olympus CX31^®^).

Blood biochemistry parameters included plasma activities of aspartate aminotransferase (AST; EC, 2.6.1.1), gamma glutamyl transferase (GGT; EC, 2.3.2.2), alkaline phosphatase (ALP; EC, 3.1.3.1), lactate dehydrogenase (LDH; EC 1.1.1.27), and creatine kinase (CK; EC, 2.7.3.2), these were analyzed by Human kits (Human^®^); and glutathione peroxidase (GPx; EC, 1.11.1.9) by Ransel (Randox^®^). All biochemical analysis were quantified using an autoanalyzer (Metrolab 2300^®^, Wiener Lab). Plasma concentration of total protein, albumin, urea, and creatinine, were analyzed using Human kits (Human^®^) and lactate using Sentinel kit (Sentinel CH^®^). Calcium (Ca) (Atomic absorption spectrometry, Thermo Electron Corporation^®^ S Serie), phosphate (P) (photometric determination with molybdate, Ultra Violet Auto analyzer Wiener lab^®^, Metrolab 2300), and Ca:P ratio was also determined.

For hematological parameters, samples were sent to a private laboratory in Santiago (Laboratorio de Química Clínica Especializada). Blood biochemistry parameters were analyzed at the Clinical Pathology Laboratory of the Universidad Austral de Chile. Finally, cortisol was determined by radioimmunoassay at the Universidad de Concepción at the Veterinary Endocrinology Laboratory.

This study was approved by the Animal Care and Use Committee of the Universidad de Chile, certificate N°19263-VET-UCH.

### Data Analysis

Descriptive statistic (mean, median, SD, minimum and maximum values), outlier analysis (Dixon–Reed and Tukey tests) and reference intervals (RI) were established with the software Reference Value Advisor (12), according to recommendations provided by the International Federation of Clinical Chemistry Clinical (IFCC) and the Clinical Laboratory Standards Institute (CLSI) (1, 2). Outliers (including anomalous values) were identified with Dixon-Reed and Tukey test, “suspected” values were conserved, while values classified as outliers were eliminated from the analysis if they were aberrant observations (biologically implausible) or retained if they were credible, in line with the American Society of Veterinary Clinical Pathology (ASVCP) recommendations for retaining rather than deleting outliers (1). Determinations of RI were generated using non-parametric methods as recommended by the ASVCP for sample sizes of at least equal to 120 individuals.

The individual values of each variable obtained for mules were assessed in relation to the established reference intervals published for Chilean urban working horses (4), working horses in Pakistan (6), domestic donkeys in the UK (3), and mules in Portugal (11). The percentage of Chilean mules with values below and above each of the reference intervals provided in the literature for horses, donkeys and mules was calculated. This was done for 27 of the 32 parameters assessed, according to the available RI for them in the literature.

The ASCVP reference intervals transference method could not be applied since the laboratories did not have a data set of at least 20 healthy reference individuals for applying this validation method.

## Results

A total of 32 hematological and serum biochemical reference intervals were established from the 142 sampled mules. As a result of damage during sampling (i.e., blood clot), anomalous results provided by the laboratory and the presence of outliers not all variables have the same sample size still all the variables remained with over 120 individuals, in accordance to the required sample size recommended by the IFCC. The variable with the minimum sample size was the N:L (*n* = 134), and maximum sample size was 142 (10 out of 32 variables) (Table 1).

**Table 1:** Hematological and biochemical reference intervals for mules in Chile, descriptive statistics, number of outliers, and 90% confidence interval for lower and upper limits of reference.

**Variable**	***N***	**Mean**	SD^*^	**Median**	Min^*^	Max^*^	RI^*^ (LRL-URL)	90% CI LRL^*^	90% CI URL^*^	**Number of outliers**
RBC count ×10^12^/L	140	7.2	0.74	7.19	5.1	8.8	6–8.5	5.1–6.1	8.4–8.8	0
Hematocrit (PCV) %	140	40	4.2	39.7	28.9	48.4	32.2–48	28.9–33.6	47.5–48.4	0
Hemoglobin g/L	140	143	16	142	99	180	113–174	99–119	168–180	0
Mean corpuscle volume (MCV) fL	140	55.4	1.7	55.5	50.9	59.7	51.9–58	50.9–52.6	58–59.7	0
Mean corpuscle volume concentration g/L	140	356	9	356	337	370	340–370	337–343	370–370	0
WBC count ×10^9^/L	137	7.7	1.3	7.7	4.5	11.2	5.3–10.8	4.5–6	10.2–11.2	3
Eosinophils ×10^9^/L	139	0.30	0.31	0.24	0	1.90	0–1.44	0–0	0.81–1.90	1
Basophils ×10^9^/L	138	31	49	0	0	0.22	0–0.16	0–0	0.13–0.22	2
Myelocytes ×10^9^/L	140	0	0	0	0	0	0	0	0	0
Metamyelocytes ×10^9^/L	140	0	0	0	0	0	0	0	0	0
Band forms ×10^9^/L	140	16	49	0	0	0.39	0–0.15	0–0	0.085–0.39	0
Neutrophils ×10^9^/L	140	4.21	1.05	4.15	1.15	7.9	2.43–7.29	1.15–2.69	5.85–7.9	0
Lymphocytes ×10^9^/L	139	3.12	0.89	3.0	1.14	5.93	1.43–5.24	1.14–1.79	4.64–5.93	1
N:L	134	1.4	0.5	1.4	0.49	2.76	0.6–2.5	0.5–0.7	2.2–2.8	5
Monocytes ×10^9^/L	139	89	0.11	64	0	0.44	0–0.36	0–0	0.34–0.44	1
Platelets ×10^9^/L	138	147.8	34.8	146.0	75	272	86.4–225.2	75–101	202–272	2
Erythrocyte sedimentation rate (mm/h)	140	44.8	21	39.5	13	104	16–95.2	13–19	83–104	0
Cortisol (nmol/L)	142	301.2	96.8	291.3	104.9	601.8	121.1–536.5	105.1–139.3	457.9–601.9	0
Albumin (g/L)	142	38.6	3.4	34	24	48	31–44.4	24–33	44–48	0
GPx IU/g Hb	137	179.9	39.7	273	106	273	110.5–268.8	106–123	255–273	0
Potassium mmol/L	142	5	0.6	4.9	2.64	6.89	3.4–6.4	2.6–3.9	5.8–6.9	0
Urea mmol/L	141	6.7	0.8	6.7	3.9	8.6	5.2–8.4	3.9–5.6	8.1–8.6	1
ALP IU/L	141	422.9	137.9	384	199	951	230–774.1	199–247	730–951	1
Proteins g/L	142	77.3	5.5	77.5	54	93	65.6–88.9	54–69	86–93	0
LDH IU/L	142	423.2	127.8	409	145	793	216.6–761.6	145–237	627–793	0
GGT IU/L	141	23.1	9.2	22	9	75	11.1–58.2	9–14	34–75	1
Phosphate mmol/L	142	29.3	6.4	25.8	16.1	45.8	16.1–45.1	16.1–19.3	38.7–45.1	0
Creatinine umol/L	142	100.9	20.6	103	38	148	46.9–140.9	38–67	131–148	0
CK IU/L	141	177.7	84.2	158	52	495	72.9–455.1	52–84	350–495	1
Calcium mmol/L	142	3	0.3	3	2.3	3.6	2.4–3.5	2.3–2.5	3.5–3.6	0
AST IU/L	142	285.1	54.9	292	138	431	142.6–390.1	138–181	369–431	0
Ca:Pi	142	3.7	0.8	3.5	1.9	6.3	2.4–6.0	1.9–2.2	5.0–5.6	0

* *SD, standard deviation; Min, minimum value; Max, maximum value; RI, reference interval; LRL, lower reference limit; URL, upper reference limit*.

Descriptive statistics, reference intervals (2.5 and 97.5 percentiles) with 90% of confidence intervals, final sample size and number of outliers for the 32 hematological and biochemical parameters are presented in Table 1. Differences in the reference intervals between Chilean mules and those established for Portuguese mules (11), donkeys from the UK (3), Pakistani working horses (6), and Chilean working horses (4) are shown in Table 2. From the 32 reference intervals obtained for Chilean mules, 27 were compared with the reference intervals provided for the above-mentioned studies, for the remaining five it was not possible because the above-mentioned studies did not report reference intervals for those variables (Myelocytes, Metayelocytes, Band forms, Erythrocyte sedimentation rate, and Cortisol). In general, RI obtained for Chilean mules were narrower than the RI provided for Portuguese mules (11), donkeys from the UK (3), Pakistani working horses (6), and Chilean working horses (4), and the variables with the most similar RI among all the studied mentioned above was for lymphocytes, neutrophils, and mean corpuscle volume concentration.

**Table 2:** Reference intervals (RI) determined for Chilean mules and those established for mules in Portugal, donkeys in the UK, working horses in Pakistan, and working horses in Chile.

**Variable**	**Reference intervals**				
	**Portugal mules^**a**^**	**Donkeys UK^**b**^**	**Pakistani working horses^**c**^**	**Chilean working horses^**d**^**	**Chilean mules**
RBC count ×10^12^/L	7.6–10.2	4.4–7.1	4.97–8.18	4.64–9.09	6–8.5
Hematocrit (PCV) %	32–43	27–42	25.0–40.3	25–46	32.2–48
Hemoglobin g/L	110.5–138	89–147	89–139	83.2–148.1	113–174
Mean corpuscle volume (MCV) fL	43.4–52.2	53–67	43.9–55.6	40–56.9	51.9–58
Mean corpuscular hemoglobin concentration g/L	339–366	310–370	331–369	–	340–370
WBC count ×10^9^/L	7.6–10.2	–	5.72–13.71	4.31–12.9	5.3–10.8
Eosinophils ×10^9^/L	–	0.1–0.9	0.00–1.13	0.07–1.19	0–1.44
Basophils ×10^9^/L	–	0–0.066	–	–	0–0.16
Neutrophils ×10^9^/L	–	2.4–6.3	2.22–7.25	1.31–8.07	2.43–7.29
Lymphocytes ×10^9^/L	–	2.2–9.6	1.44–6.78	1.23–7.19	1.43–5.24
N:L	–	–	–	0.45–4.74	0.6–2.5
Monocytes ×10^9^/L	–	0–0.75	0–0.62	0.060–0.779	0–0.36
Platelets ×10^9^/L	–	95–384	85–276	71–422	86.4–225.2
Albumin (g/L)	–	21.5–31.6	18–28	21–50	31–44.4
GPx IU/g Hb	–	–	–	11–278	110.5–268.8
Potassium mmol/L	–	3.2–5.1	3.3–4.9	–	3.4–6.4
Urea mmol/L	–	1.5–5.2	3.6–11.4	3.73–10.9	5.2–8.4
ALP IU/L	–	98–252	59–319	108–565	230–774.1
Total proteins g/L	–	58–76	57–76	43–91	65.6–88.9
LDH IU/L	536–737	–	–	353–1746	216.6–761.6
GGT IU/L	15–29.5	14–69	11–32	7–112	11.1–58.2
Phosphate mmol/L	0.73–1.09	–	0.39–1.64	0.5–5.3	16.1–45.1
Creatinine umol/L	0.88–1.20	53–118	61.3–133.0	53.9–134.3	46.9–140.9
CK IU/L	184–285	128–525	123–358	107–821	72.9–455.1
Calcium mmol/L	–	2.2–3.4	2.68–3.14	2–12.4	2.4–3.5
AST IU/L	152–204	238–536	189–456	100–732	142.6–390.1
Ca:Pi	–	–	–	1.3–6.7	2.4–6.0

a *Reference intervals from McLean et al. (11)*.

b *Reference intervals from Burden et al. (3)*.

c *Reference intervals from Pritchard et al. (6)*.

d *Reference intervals from Aros et al. (4)*.

Table 3 shows the percentage of Chilean mules that presented variables above or below the RI provided in the literature. The results indicate that when using RI obtained for Portuguese mules for all the variables mules below and above the RI were found. On the other hand, when using the RI reported for Chilean Working horses, mules below or above the RI were only found for 11 of the 25 RI available. The biggest differences, more than 80% of Chilean mules outside literature RI, were found for MCV, LDH and AST in relation to Portuguese mules; albumin, urea and ALP for UK donkeys; and albumin and ALP for Pakistani working horses.

**Table 3:** Percentage of individual Chilean mules that have values that fall below or above the reference intervals established in the literature for mules in Portugal (11), Donkeys in the United Kingdom (UK) (3), working horses in Pakistan (6), Chilean working horses (4).

**Variable**	**Portugal mules**		**UK donkeys**		**Pakistani working horses'**		**Chilean working horses'**	
	**% Below RI**	**% Above RI**	**% Below RI**	**% Above RI**	**% Below RI**	**% Above RI**	**% Below RI**	**% Above RI**
RBC count ×101^2^/L	67.1	0	0	0	0	13.5	0	0
Hematocrit (PCV) %	1.4	20.7	0	29.2	0	44.2	0	10
Hemoglobin g/L	1.4	59.2	0	37.1	0	57.1	0	33.5
Mean corpuscle volume (MCV) fL	0	97.8	9.2	0	0	47.1	0	24.2
Mean corpuscular hemoglobin concentration g/L	1.4	16.4	0	0	0	8.5	–	–
WBC count ×10^9^/L	46.7	4.3	–	–	2.9	0	0	0
Eosinophils ×10^9^/L	0	33.1	27.2	5.1	0	3.6	17.6	3.6
Basophils ×10^9^/L	–	–	0	28.7	–	–	–	–
Neutrophils ×10^9^/L	–	–	2.1	3.5	0.7	2.1	0	0
Lymphocytes ×10^9^/L	–	–	14.3	0	2.1	0	15.1	0
N:L	–	–	–	–	–	–	0	0
Monocytes ×10^9^/L	–	–	0	0	0	0	47.4	0
Platelets ×10^9^/L	–	–	3.6	0	1.4	0	0	0
Albumin (g/L)	–	–	0	97.1	0	98.5	0	0
GPx IU/g Hb	–	–	–	–	–	–	0	0
Potassium mmol/L	–	–	1.4	38	1.4	52.8	0	0
Urea mmol/L	–	–	0	97.1	0	0	0	0
ALP IU/L	–	–	0	94.3	0	82.2	0	12.7
Total proteins g/L	–	–	0	59.1	0	59.1	0	0.7
LDH IU/L	81.7	2.8	–	–	–	–	32.3	0
GGT IU/L	8.5	11.3	8.5	0.7	2.12	8.5	0	0
Phosphate mmol/L	17.6	7.04	–	–	0	0	0	0
Creatinine umol/L	23.2	14.1	2.1	15.4	2.8	4.2	2.8	2.1
CK IU/L	66.6	11.3	26.2	0	24	4.9	12	0
Calcium mmol/L	–	–	0	5.6	6.3	33.1	0	0
AST IU/L	4.9	92.2	14.7	0	6.3	0	0	0
Ca:Pi	–	–	–	–	–	–	0	0

## Discussion

In the present study hematological and biochemical reference intervals for healthy mules were established. Although previous studies have evaluated blood parameters in mules, these have included small sample sizes (*n* = 20) (9, 11), providing limited information about normal hematological and biochemical values, since they do not comply with international recommendations (1, 2). To the authors knowledge, this is the most extensive evaluation of blood parameters in order to establish reference intervals for mules. This considering that it was possible to assess a population of mules greater than the minimum sample size of at least 120 healthy individuals recommended by the IFCC and ASVCP reference interval guidelines for veterinary species (1, 2) in order to obtain reliable information.

ASVCP reference intervals guidelines recommend to validate the proposed RI with at least 20 healthy reference individuals from the laboratory's animal patient population, using the same laboratory methods for an acceptable transference comparison (1). However, none of the three laboratories where blood parameters were analyzed had data from healthy mules. Therefore, we compared previous reference intervals established for horses, donkeys and mules with those obtained for Chilean mules in order to identify similitudes and differences between Chilean mules and other equids. In view that the analytic method across countries are not always the same, caution must be taken in comparisons between studies. In the same line, the fact that it is difficult to access this type of information for this hybrid highlights the importance for calculating reference intervals.

The current study found that the RI's obtained for the Chilean mule population were similar to those reported for Chilean working horses (4). These similarities may partly be explained by the influence exerted by geographical location on the nutrients provided by the forage that Chilean horses and mules have available and to biological factors, such as the genetic influence of mares. It needs to be taken into consideration that the Chilean mules sampled are produced in the army with local crosses of mixed breed mares. On the other hand, both studies used the same laboratories to process the samples and these results could be reflecting the instrument and techniques used to generate the RI. The major difference between Chilean mules and Chilean working horses was the lower concentration of monocytes observed in the mules evaluated (47.4% of mules had fewer monocytes than Chilean working horses), this difference could be associated to the fact that the presence of chronic inflammatory conditions are commonly reported for working horses worldwide (13–15).

The main differences found between RI's established for Chilean mules and those for UK donkeys and Pakistani working horses were related with higher values of albumin and ALP for Chilean mules. Also, Chilean mules presented higher concentrations of urea than the UK donkeys. Serum concentration of albumin, ALP and urea could reflect the effects of diet, since working equids often have malnutrition (6). On the other hand, donkeys have a higher capacity of urea recycling than horses (16), especially when fed low protein diet, and may have better digestive efficiency (17). Furthermore, mules are thought to have a digestive system more similar to donkeys than horses, including an enhanced urea recycling ability (18). Hence diet provided to Chilean mules could be the cause of the higher urea concentration, considering that donkeys sampled by Burden et al. (3) were mainly fed with straw. Straw has a lower content of protein than alfalfa hay (19), the latter being the main source of forage provided to the mules sampled in this study.

One interesting finding is that the RI's calculated for Portuguese mules (11) were very different from the results obtained for Chilean mules, considering that all variables were below or above the RI provided (Table 1). Mules are the result of crossing a mare (female horse) and a jack (male donkey), resulting in important genetic variations in the offspring, according to the parents breed. The differences in the breeds used across countries to obtain mules may have an influence on their biological features complicating the possibility of applying the same RI's between mules with different genetic background. In the case of the Chilean army mainly cross breed mares are used and jack's breed vary (Baudet du Poitou, mixed Poitou breed, and local cross breed). Furthermore, in accordance with ASVCP reference interval guidelines there is a higher degree of uncertainty when using a small sample size (1), thus the differences could be the result of the small sample size used by McLean et al. (11) (*n* = 20) resulting in a minor accuracy in the measure of the RI. In order to minimize this effect, the RI should be calculated by robust methods if normality cannot be established (1). In the case of the study of Portuguese mules only means and standard deviations are provided for each variable (11).

Selenium deficiency has been reported in horses from several countries and breeds, associated with clinical cases of nutritional miodegeneration and steatosis (20–22), nevertheless there are no serum GSH-Px reference values for mules that allow to determine the existence of selenium deficiency. According to reference values provided by Wittwer (2) for Chilean equines, serum GSH-Px values must be above 130 IU/g Hb. In this study 8.7% of mules were below this reference. Weber (23) studied the GPx activity in mules and horses at birth and 30 days after, they found that mules had higher levels of GPx activity than horses at both sampling times, however concentrations did not reach the lowest level established by Wittwer (2). In a recent study, Bazzano et al. (24) found that donkeys had a lower range of serum selenium than horses, and suggested that donkeys may have a lower selenium requirement. Limited comparisons can be made to establish a selenium deficiency in mules and it would be inappropriate to compare the RI established for this population with others.

Cortisol has been widely used to measure stress in horses (25, 26) and in lesser extent in donkeys (27). To our knowledge there are no published data on cortisol RI for mules, in order to compare them with the present results. Wittwer (2) provides a reference value for cortisol between 32 and 240 nmol/L, which is lower than the reference interval estimated in this study (120.5–473.6 nmol/L), especially considering that mule's mean cortisol concentration falls above the reported upper limit for horses. Dugat et al. (28) found that cortisol values overlapped between donkeys and horses and ranged from 66.2 to 165.5 nmol/L, values that fall within the interval provided by Wittwer (2) for horses. The reasons for the higher concentration of cortisol in mules is not clear, but plasma cortisol can be altered for a multitude of reasons, some of them are normal (i.e., exercise), and others pathological situations (i.e., chronic disease, blood sampling, stressful managements, among others) (29).

The neutrophil:lymphocyte ratio (N:L) established in this study falls within values provided for Chilean working horses, but the reference interval established for the Chilean mules was narrower and with a lower upper reference limit. This could be explained because working horses tend to have constant deprivation of their basic needs and are commonly overworked, and in consequence chronically stressed (13); by contrast, Chilean army mules have their basic needs fulfilled and are subjected to more predictable husbandry routines.

This study provided the first comprehensive assessment of RIs for mules and identified important differences between the RIs established for mules and those established for horses and donkeys. These findings provide support of the necessity to have specific RIs for mules, highlighting the importance of using appropriate references for each population. This would allow improving clinical diagnosis and treatment decision-making for mules with different husbandry conditions and biological features.

The population used in this study lives in a steppe region of Chile, has very homogeneous housing conditions, diet, genetic background and sporadic work, so caution must be taken to use this RIs with mules located in tropical and subtropical areas subjected to heavy working labors. The RIs obtained in this study will provide support to the veterinary services of the Chilean army, improving health monitoring and welfare assessment of mules. Further research is required to fully understand physiological differences between mules and other type of equids, and the influence of their biological background, husbandry conditions, environmental factors and the work performed.

## Data Availability Statement

The datasets generated for this study are available on request to the corresponding author.

## Ethics Statement

The animal study was reviewed and approved by Animal care and use committee, Universidad de Chile. Certificate number 19263-VET-UCH.

## Author Contributions

JL and TT conceived and designed the study. JL organized and took the blood samples, performed clinical examination, and analyzed the data. JL and TT wrote the manuscript.

### Conflict of Interest

The authors declare that the research was conducted in the absence of any commercial or financial relationships that could be construed as a potential conflict of interest.
